# Comparison of CRT and LCD monitors for objective estimation of visual acuity using the sweep VEP

**DOI:** 10.1007/s10633-022-09883-x

**Published:** 2022-07-05

**Authors:** Torsten Straßer, Denise Tara Leinberger, Dominic Hillerkuss, Eberhart Zrenner, Ditta Zobor

**Affiliations:** 1 Institute for Ophthalmic Research, Centre for Ophthalmology, University of Tuebingen, Elfriede-Aulhorn-Str. 7, 72076 Tübingen, Germany; 2 University Eye Hospital Tuebingen, Centre for Ophthalmology, University of Tuebingen, Tübingen, Germany; 3 Werner Reichardt Centre for Integrative Neuroscience (CIN), University of Tuebingen, Tübingen, Germany; 4 Department of Ophthalmology, Semmelweis University Budapest, Budapest, Hungary

**Keywords:** Visual evoked potentials, Visual acuity estimation, Sweep VEP, Stimulator, LCD, CRT, Monitor

## Abstract

**Purpose:**

To investigate the applicability of liquid crystal displays (LCD) as suitable replacement for cathode ray tube monitors (CRT) as stimulator for the sweep VEP for estimating visual acuity.

**Methods:**

In a first experiment, sweep VEPs were recorded in 13 healthy volunteers with best-corrected visual acuity with an LCD and a CRT monitor, respectively. Time-to-peak after stimulus and peak-to-trough amplitudes as well as the visual acuity, estimated using a second-order polynomial and the modified Ricker model, were compared between both monitor types. In a second experiment, sweep VEPs were recorded in six healthy volunteers with two levels of stimulus contrast using artificially reduced visual acuities as well as best-corrected with the same monitors as in the first experiment and additionally, a modern LCD gaming monitor with a response time of 1 ms. Time-to-peak after stimulus and peak-to-trough amplitudes were compared between the different combinations of monitors and contrasts. Finally, visual acuities estimated using the modified Ricker model were compared to subjective visual acuities determined using the Freiburg Visual Acuity and Contrast Test (FrACT).

**Results:**

In the first experiment, the time-to-peak after stimulus presentation was statistically significantly delayed for LCD displays (mean difference [confidence interval]: 60.0 [54.0, 65.9] ms; *t*(516) = 19.7096, *p* < 0.0001). Likewise, peak-to-trough amplitudes were statistically significantly smaller for the LCD stimulator, however, not clinically relevant (mean difference [confidence interval]: − 0.89 [– 1.59, − 0.20] µV; *t*(516) =  − 2.5351, *p* = 0.0115). No statistically significant effect of the monitor type on the estimated visual acuity was found for neither method, second-order polynomial, nor the modified Ricker model. In the second experiment, statistically significant delays of the time-to-peak after stimulus onset were found for all combinations of monitor and contrast compared to the CRT monitor. A statistically significant, but not clinically relevant, difference of the peak-to-trough amplitudes was only found between the CRT monitor and the LCD gaming monitor (mean difference [confidence interval]: 2.6 [1.2, 4.0] µV; *t*(814) = 4.66, *p* < 0.0001). Visual acuities estimated from LCD stimulation significantly underestimated the subjective visual acuity up to 0.2 logMAR using the conversion formula of the first experiment. No statistically significant difference was found when using conversion formulas adjusted for each combination of monitor and contrast.

**Conclusions:**

Based on the results of this study, LCD monitors may substitute CRT monitors for presenting the stimuli for the sweep VEP to objectively estimate visual acuity. Nevertheless, it is advisable to perform a calibration and to collect normative data of healthy volunteers using best-corrected and artificially reduced visual acuity for establishing a conversion formula between sweep VEP outcome and the subjective visual acuity before replacing a CRT with an LCD stimulator.

**Supplementary Information:**

The online version contains supplementary material available at 10.1007/s10633-022-09883-x.

## Introduction

Despite the developments in display technology in recent years like liquid crystal (LCD), thin-film transistor (TFT), or organic light-emitting diode (OLED) displays, cathode ray tube (CRT) monitors are still the method of choice for eliciting visual evoked potentials (VEP) in most laboratories. This is because a reliable measurement of VEPs requires a constant mean luminance during the stimulation, which is easily achieved with CRT but not with LCD monitors, which typically present a brief so-called luminance artifact during pattern reversal [1]. However, CRT monitors are now rarely available in stores, and only used ones can be bought on webshops like eBay. Since the phosphors of CRT displays, used to produce the image on the monitor, have a lifespan of 5–10 years [2], alternatives will be required now or in the near future. Several studies have compared the applicability of LCD for pattern VEP stimulus presentation and found significant delays in the P100 latency from about 8 ms [2, 3] up to more than 25 ms [3, 4]. However, Nagy et al. found no differences between N75 and P100 amplitude obtained using CRT and LCD stimulators [2]. Since for the estimation of the visual acuity using VEP [5], only the VEP amplitudes in response to patterns with varying frequencies are analyzed either directly [6, 7] or using Fourier analysis [8], and the recently published ISCEV extended protocol for VEP methods of estimation of visual acuity recommends only to ensure the absence of luminance artifacts caused from non-CRT displays [9], we investigated the applicability of an LC-display as a suitable replacement for CRT monitors as a stimulator for the sweep VEP. In a first experiment, we compared visual acuities estimated from sweep VEPs presented on a CRT to those presented on an LCD monitor in a cohort of healthy volunteers with best-corrected visual acuity. In a second experiment, we additionally compared visual acuities estimated from sweep VEPs in a smaller cohort using artificially reduced visual acuities as well as best-corrected, in addition using a modern LCD gaming monitor with two different levels of contrast.

## Methods

### Participants

For the first experiment, thirteen healthy volunteers, aged between 24 and 64 years (mean 36.6 ± 11.5 SD), were recruited from the staff of the Centre for Ophthalmology at the University of Tuebingen according to the following inclusion criteria: no ocular or systemic pathology, no abnormalities in a general ophthalmic examination, best-corrected visual acuity (BCVA) of 0.8 (decimal) or better, and normal VEP, according to the ISCEV standard [1]. Subjective visual acuity was measured best corrected with Snellen targets presented using a chart projector (Chart Projector CP-500, Shin-Nippon, Japan). The second experiment was performed with six healthy volunteers, aged between 20 and 43 years (mean 28.9 ± 8.7 SD), recruited from the staff of the Center for Ophthalmology at the University of Tuebingen (one volunteer also took part in the first study), according to the same criteria as for the first experiment. Visual acuities were determined using the Freiburg Visual Acuity & Contrast Test (FrACT) [10, 11] for BCVA and two artificially reduced viewing conditions using Bangerter occlusion foils (0.6, 0.4) [12]. In both experiments, only one eye of each participant was assessed.

### Sweep VEP Stimulation and Recording

Sweep VEPs were recorded monocularly with an Espion e^2^ (Diagnosys LLC, Cambridge, UK) electrophysiological recording system at a sampling frequency of 1000 Hz using Gold-cup electrodes, mounted on *Fz* (reference), *Oz* (active), and *Cz* (ground) according to the International 10–20 System [13] and the ISCEV standard [1]. Impedances were checked before the start of the recording and kept well below 5 kΩ.

In both experiments, checkerboards with logarithmically equidistant increasing dominant spatial frequencies (0.6, 0.9, 1.4, 2.1, 3.3, 4.9, 7.3, 10.4, 18.2, 24.4, and 36.5 cpd, calculated according to Fahle and Bach [14]) were used as stimuli. Each checkerboard was presented for 50 ms, followed by an equiluminant uniform gray background of 300 ms [7]. During the first experiment, the sequence was repeated two times [1], with a short break after the first cycle. Fifty traces of each cycle were averaged to increase the signal-to-noise ratio. For the LCD stimulators in the second experiment, only one sequence with 10 sweeps free of artefacts was recorded and averaged.

During the first experiment, the checkerboard stimuli were generated using a custom Java™-based software with a Michelson contrast of (≥ 90% (measured at intermediate spatial frequencies) and a space-averaged mean luminance of ~ 30 cd/m^2^, determined with an LS-100 luminance meter (Konica Minolta, Tokyo, Japan). The sweep VEP was recorded first using stimuli presented on a 21″ CRT monitor (Model V999, Elonex, Birmingham, UK; resolution: 1600 × 1200 pixel; frame rate: 60 Hz) and in a second run (between 8 to 24 min later) on a 21″ TFT color LCD monitor (ColorEdge CG21, EIZO Corporation, Japan; resolution: 1600 × 1200 pixel; response time: 50 ms; screen refresh rate: 60 Hz).

For the second experiment, in addition to the same monitors as in the first experiment (CRT, LCD old), a modern gaming monitor (25″, Alienware AW2518HF, Dell Inc., Round Rock, USA; resolution 1920 × 1080 pixel; response time: 1 ms; screen refresh rate: 60 Hz) was used (LCD new). Sweep VEPs recorded with the CRT monitor used the same software and setup as in the first experiment, whereas for the LCD monitors, stimuli were generated using a custom stimulus paradigm implemented using PsychoPy (Version 2021.1.4) [15, 16] with each of two Michelson contrasts 90% and 70%. Sweep VEPs were recorded with best-corrected visual acuity and with artificially reduced visual acuity.

In all cases, stimuli were presented synchronously to a trigger sent from the Espion e^2^ system.

### Sweep VEP Analysis and Estimation of Objective Visual Acuity

Peaks and troughs of the averaged traces were determined using a multi-scale-based peak detection algorithm [17] implemented in ERG Explorer [18], verified and manually corrected if necessary, and exported along with the corresponding spatial frequencies.

The objective visual acuity was estimated with an online tool (https://strator1.github.io/SweepVEP) using the spatial frequency at the maximum amplitude (sf_max_) derived from the fitted modified Ricker function [7] (first and second experiment), and the limiting spatial frequency (sf_limiting_) of a second-order polynomial fit [5, 7] (first experiment only).

### Statistical Analysis

#### First Experiment

To evaluate a possible effect of the monitor type used for stimulation on the sweep VEP recorded during the first experiment linear mixed-effects models, fit by restricted maximum likelihood estimates (REML) were used to assess the significance of the categorical predictor variables monitor type (*m* = {LCD, CRT}), spatial frequency of the stimulation pattern, and recording cycle (*r* = {1, 2}), as well as their interactions in explaining variations in the dependent variable (*Y* = {*t*_D_, *a*_PT_}) times-to-peak after stimulus onset (*t*_D_), and the corresponding peak-to-trough amplitude (*a*_PT_), respectively. To account for repeated measurements and for the inter-individual variability, the volunteer (ID) was set as a random effect (Eq. 1).

1 $${Y}_{\mathrm{ijkl}}=\mu +{\mathrm{ID}}_{\mathrm{i}}+{m}_{\mathrm{j}}+{\mathrm{sf}}_{\mathrm{k}}+{r}_{\mathrm{l}}+{\left(m\times \mathrm{sf}\right)}_{\mathrm{jk}}+{\left(m\times r\right)}_{\mathrm{jl}}+{\left(\mathrm{sf}\times r\right)}_{\mathrm{kl}}+{\left(m\times \mathrm{sf}\times r\right)}_{\mathrm{jkl}}+{\varepsilon }_{\mathrm{ijkl}}.$$

Limits-of-agreement (LoA) [19] and intraclass correlation coefficients (ICC(3, 1): single measure, two-way mixed, absolute agreement) between objective visual acuities estimated from stimulation with an LCD and a CRT monitor, respectively, were calculated using both the second-order polynomial and the modified Ricker model after ensuring normality and homogeneity of variances.

Descriptive statistics for the differences between subjective and estimated visual acuities were calculated and mean differences were compared with a hypothetical difference of 0 logMAR using one-sample *t* tests after confirming a normal distribution of the data. A correction for multiple comparisons was omitted in favor of higher sensitivity for possible statistically significant differences.

Possible effects of the monitor type used for stimulation on the differences between subjective and estimated visual acuities were evaluated by fitting a linear mixed-effects model (Eq. 2) with the difference as dependent variable (*Y*), monitor type (*m* = {LCD, CRT}) and recording cycle (*r* = {1, 2}) as predictor variables, and volunteer (ID) as a random effect to account for repeated measurements.

2 $${Y}_{\mathrm{ijk}}=\mu +{\mathrm{ID}}_{i}+{m}_{j}+{r}_{k}+{\left(m\times r\right)}_{\mathrm{jk}}+{\varepsilon }_{\mathrm{ikl}}.$$

#### Second Experiment

The effects of the spatial frequency (sf), the stimulation type (*s* = {CRT/high contrast, LCD old/low contrast, LCD old/high contrast, LCD new/low contrast, LCD new/high contrast}) and the viewing condition (*c* = {sc, 0.6, 0.4}), as well as their interaction with the dependent variables amplitude and time-to-peak after stimulus onset (*Y* = {*t*_D_, *a*_PT_}), were evaluated using linear mixed-effects models (Eq. 3).

3 $${Y}_{\mathrm{ijkl}}=\mu +{\mathrm{ID}}_{i}+{s}_{j}+{\mathrm{sf}}_{k}+{c}_{l}+{\left(s\times \mathrm{sf}\right)}_{\mathrm{jk}}+{\left(s\times c\right)}_{\mathrm{jl}}+{\left(\mathrm{sf}\times c\right)}_{\mathrm{kl}}+{\left(s\times \mathrm{sf}\times c\right)}_{\mathrm{jkl}}+{\varepsilon }_{\mathrm{ijkl}}.$$

The least square means of time-to-peak and peak-to-trough amplitude between the CRT monitor with high contrast as control and the other combinations of monitor and contrast were compared using post hoc Dunnett’s tests.

Subsequently, the spatial frequency of the maximum amplitude (sf_max_) and the signal-to-noise level (SNR) were calculated by fitting the modified Ricker function [7]. The SNR was compared between the different combinations of monitor and contrast using Tukey–Kramer honestly significant difference (HSD) tests. sf_max_ was transformed into the estimated visual acuity, first based on the formula published in [7], and second, by calculating a new relation to the subjective visual acuity, and the mean differences to the subjective visual acuity were compared with a hypothetical difference of 0 logMAR using one-sample *t* tests after confirming a normal distribution of the data. Before utilizing the results of the linear mixed-effects models for further statistical inference, the variance inflation factors (VIF) of the predictor variables were calculated and assured to fall well below the common threshold value, indicating no collinearity between them [20]. Furthermore, the model residuals were confirmed visually to be normally distributed, while homoscedasticity (homogeneity of the residual variances) was tested using the Brown-Forsythe test [21] and reported in case of violations.

Statistical analyses were carried out using JMP 15.2 (SAS Institute Inc., Cary, NC, USA). ICCs were calculated using SPSS Version 27 (IBM Corp., Armonk, NY, USA).

## Results.

### First Experiment

**Fig. 1:**
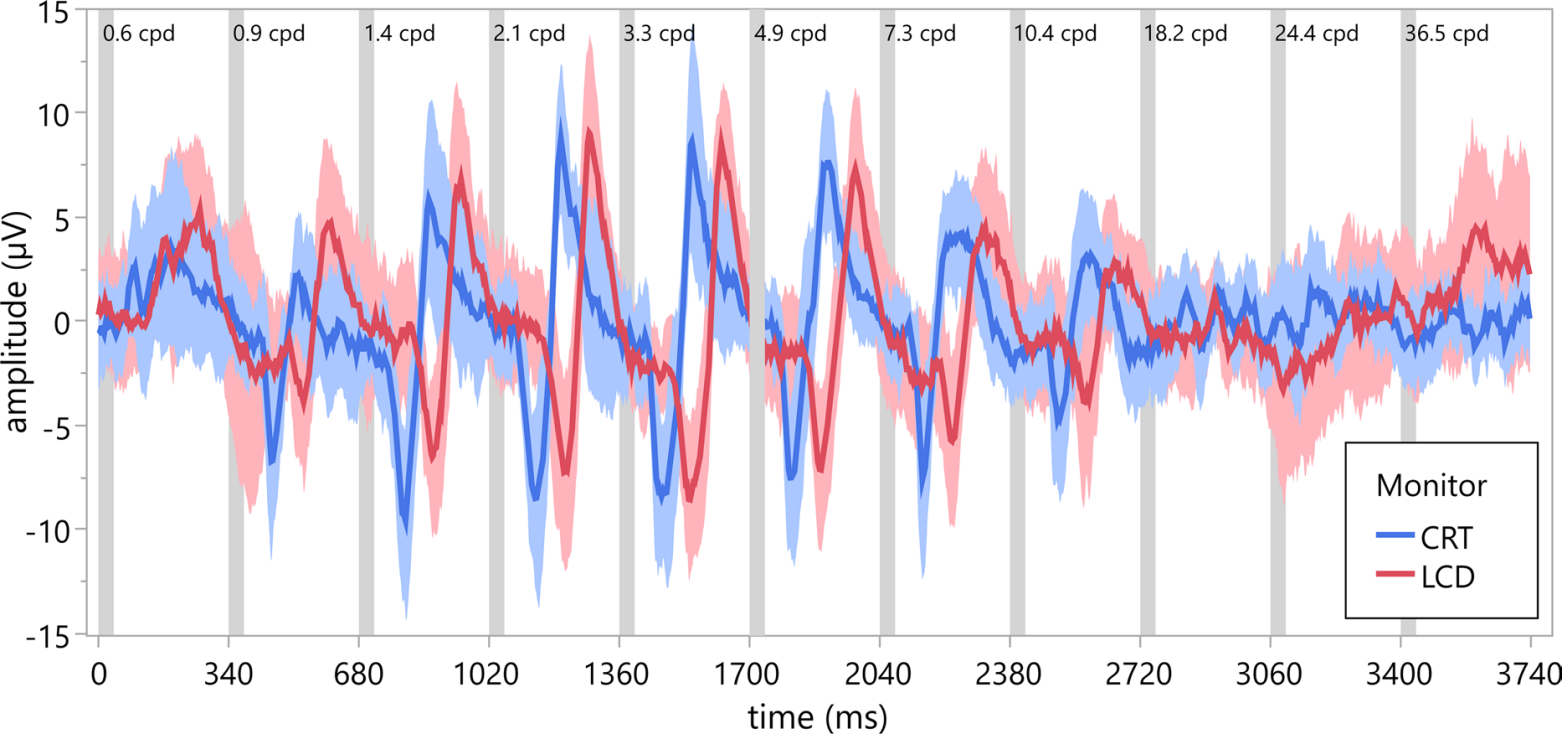
Grand average (mean and standard deviation, shaded areas) of the first cycle of the Sweep VEP of 13 healthy volunteers of the first experiment to repeated stimulation with pattern onset stimulation of increasing spatial frequency (40 ms onset, 300 ms offset, isoluminant, 11 spatial frequencies) presented first, on a CRT (blue), and second, on an LCD (red) monitor. Responses recorded using the LCD monitor are markedly delayed

Figure 1 depicts the grand average of the first recording cycle of the 13 volunteers (average of the mean of 50 traces per volunteer). The sweep VEP amplitudes in response to stimulation using the LCD monitor (red traces) are consistently delayed compared to those using the CRT monitor (blue traces) in all volunteers. Data of the second recording cycle (not shown) show a corresponding pattern. Individual sweep VEPs of the first and the second recording cycle are available in Online Resource 1.

The residuals of both models, with the dependent variables time-to-peak after stimulus onset and peak-to-trough amplitude, respectively, followed a normal distribution. However, Brown-Forsythe tests revealed heteroscedasticity (time-to-peak: *F*(1, 570) = 7.17, *p* = 0.0076; peak-to-trough amplitude: *F*(1, 570) = 70.20, *p* < 0.0001).

**Fig. 2:**
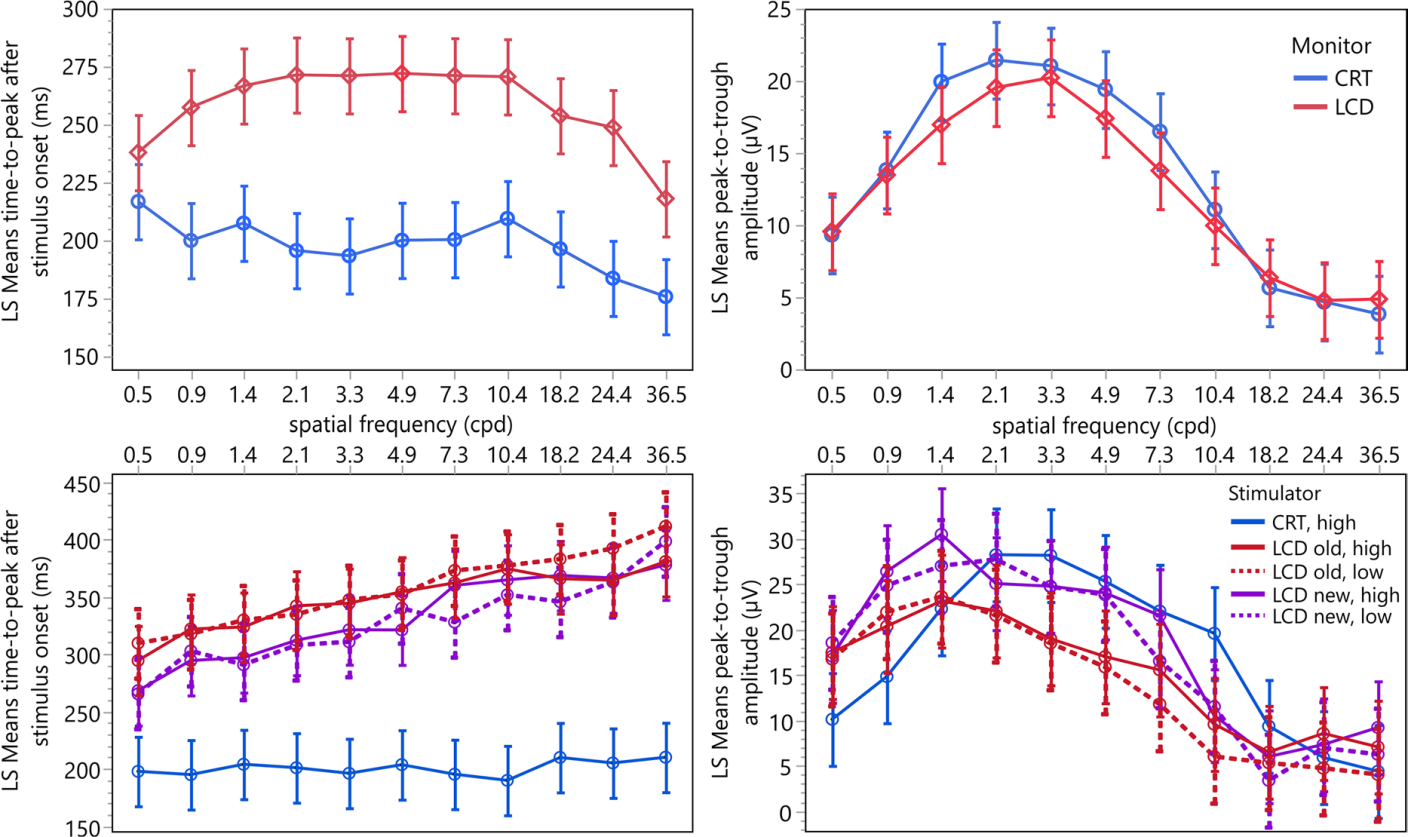
Least square means and confidence intervals (whiskers) of the models’ variables (left: time-to-peak after stimulus onset, *t*_P_; right: peak-to-trough amplitude, *a*_PT_) for the interaction between the spatial frequency of the stimulation pattern and the stimulator type used for the stimulation (upper panel: CRT: blue; LCD: red; lower panel: CRT/high contrast: solid blue, LCD old/high contrast: solid red, LCD old/low contrast: dotted red, LCD new/high contrast: solid purple, LCD new/low contrast: dotted purple). Note the different scales

The upper panels of Fig. 2 depict the least square means for the interaction between the spatial frequency and the monitor type for the time-to-peak after stimulus onset and the peak-to-trough amplitude. Again, the data for the LCD monitor are shown in red and those for the CRT monitor in blue.

Both models revealed statistically significant effects of the spatial frequency of the stimulation pattern and the monitor type used for stimulation. For predicting time-to-peak, an additional statistically significant interaction between spatial frequency and monitor type was found. No statistically significant effects were found for the recording cycle or any of its interactions. The effects and their interactions of both models are listed in Table 1.

**Table 1:** Results of the linear mixed-effects models with the dependent variables time-to-peak after stimulus onset *t*_D_ and the corresponding peak-to-trough amplitude *a*_PT_

	Time-to-peak after stimulus onset (*t*_D_) *n* = 572, *R*^2^_adj_ = .73		Peak-to-trough amplitude (*a*_PT_) *n* = 572, *R*^2^_adj_ = .75	
Effect	*F*-Statistic	*p* value	*F*-Statistic	*p* value
*m*	*F*(1, 516) = 388.4692	< 0.0001***	*F*(1, 516) = 6.4265	0.0115*
*r*	*F*(1, 516) = 2.8273	0.0933	*F*(1, 516) = 0.6072	0.4362
sf	*F*(10, 516) = 5.9525	< 0.0001***	*F*(10, 516) = 113.8292	< 0.0001***
*m* x *r*	*F*(1, 516) = 0.9432	0.3319	*F*(1, 516) = 0.1925	0.6610
*m* x sf	*F*(10, 516) = 2.6358	0.0039**	*F*(10, 516) = 1.3703	0.1906
*r* x sf	*F*(10, 516) = 1.0337	0.4134	*F*(10, 516) = 0.3800	0.9553
*m* x *r* x sf	*F*(10, 516) = 0.4309	0.9315	*F*(10, 516) = 0.2409	0.9920

*m* monitor type, *r* recording cycle, *sf* spatial frequency

Alpha level = 0.05; asterisks indicate the level of significance: *p < 0.05, **p < 0.01, ***p < 0.001

Post hoc comparisons using two-tailed *t* tests indicated statistically significant mean differences of the least square means (± SE) of the time-to-peak *t*_P_ (CRT = 198.2 ± 4.6 ms, LCD = 258.1 ± 4.6 ms; mean difference = 60.0 [54.0, 65.9] ms; *t*(516) = 19.7096, *p* < 0.0001), and the peak-to-trough amplitude *a*_PT_ (CRT = 13.34 ± 1.04 µV, LCD = 12.45 ± 1.04 µV; mean difference [95% confidence interval] =  − 0.89 [− 1.59, − 0.20] µV; *t*(516) =  − 2.5351, *p* = 0.0115) between stimulation using LCD and CRT monitors.

The visual acuities estimated from stimulation using an LCD monitor and an CRT monitor showed a moderate agreement for both estimation methods, according the classification of Koo and Li [22] (Ricker model: ICC = 0.63, CI [0.33; 0.81], second-order polynomial: ICC = 0.59, CI [0.23; 0.78]). The limits-of-agreement were [− 0.07; 0.11] logMAR for the Ricker model and [− 0.11; 0.11] logMAR for the second-order polynomial model, respectively.

One sample *t* tests of the difference between subjective and estimated visual acuities revealed no statistically significant difference from the hypothetical difference of 0 logMAR. The descriptive statistics of the mean differences and the results of the *t* tests are given in Table 2. Brown-Forsythe tests revealed no statistically significant difference of the variances between CRT and LCD monitors for both estimation methods (second-order polynomial: *F(*1, 50) = 0.0862, *p* = 0.7703; modified Ricker function: *F*(1, 50) = 0.7633, *p* = 0.3865).

**Table 2:** Descriptive statistics of the mean difference (± SD) between subjective visual acuity and visual acuities determined using the second-order model and the modified Ricker model using a CRT and an LCD monitor for stimulation

Monitor	Sequence #	Mean difference ± SD (logMAR)	One-sample *t* test, hypothetical diff. = 0 logMAR	
			*t*-statistics	*p* value
Subjective VA—estimated VA, second-order parabola				
CRT	1	0.01 ± 0.12	*t*(12) = 0.2324	0.8201
CRT	2	0.00 ± 0.11	*t*(12) = − 0.0658	0.9486
LCD	1	− 0.02 ± 0.10	*t*(12) = − 0.6482	0.5290
LCD	2	0.02 ± 0.13	*t*(12) = 0.5334	0.6035
Subjective VA—estimated VA, modified Ricker function				
CRT	1	0.00 ± 0.09	*t*(12) = 0.1743	0.8645
CRT	2	− 0.02 ± 0.14	*t*(12) = − 0.5373	0.6009
LCD	1	− 0.02 ± 0.09	*t*(12) = − 0.9521	0.3598
LCD	2	− 0.03 ± 0.11	*t*(12) = − 1.0269	0.3247

One sample t-tests compared the differences between subjective visual acuity and estimated visual acuities with the hypothetical difference of 0 logMAR

*N* = 13 for each condition; data normality confirmed using Sharp-Wilk tests; alpha level = 0.05

Accordingly, the analysis of possible effects of the monitor type, the recording cycle, or their interaction using a linear mixed-effects model revealed no statistically significant effects on the difference between subjective and estimated visual acuity Table 3.

**Table 3:** Results of the analysis of the possible effects of the monitor type, the recording cycle, or their interaction, on the differences between subjective and estimated visual acuities using linear mixed-effects models for both methods of estimation, second-order polynomial and modified Ricker function

Effect	Difference subjective VA—estimated VA			
	Second-order polynomial *n* = 52, *R*^2^_adj_ = 0.69		Modified Ricker function *n* = 52, *R*^2^_adj_ = 0.74	
	*F*-Statistic	*p* value	*F*-Statistic	*p* value
*m*	*F*(1, 36) = 0.0067	0.9350	*F*(1, 36) = 1.2268	0.2754
*r*	*F*(1, 36) = 0.4673	0.4986	*F*(1, 36) = 0.9307	0.3411
*m* x *r*	*F*(1, 36) = 1.3445	0.2539	*F*(1, 36) = 0.2796	0.6002

*m* monitor type, *r* recording cycle

Alpha level = 0.05; asterisks indicate the level of significance: * *p* < 0.05, ** *p* < 0.01, *** *p* < 0.001

#### Second Experiment

The residuals of both models, with the dependent variables time-to-peak after stimulus onset and peak-to-trough amplitude, followed a normal distribution. However, Brown-Forsythe tests revealed heteroscedasticity (time-to-peak: *F*(1, 982) = 26.4329, *p* =  < 0.0001; peak-to-trough amplitude: *F*(1, 982) = 199.9453, *p* < 0.0001).

The lower panels of Fig. 2 depict the least square means for the interaction between the spatial frequency and the stimulator type for the time-to-peak after stimulus onset and the peak-to-trough amplitude using best-corrected visual acuity. The data for the LCD monitors are shown in red (LCD old) for the conventional monitor and purple (LCD new) for the gaming monitor, and those for the CRT monitor in blue. Solid lines indicate high contrast, dotted lines low contrast checkerboard patterns. The results for artificially reduced visual acuity are given in Online Resource 2.

Both models revealed statistically significant effects of the viewing condition and the spatial frequency of the stimulation pattern. Furthermore, statistically significant effects of the stimulation type used for the presentation of the checkerboard pattern as well as of the interactions between stimulator type and viewing condition and spatial frequency were found. Finally, for predicting the peak-to-trough-amplitude, an additional statistically significant interaction between spatial frequency and viewing condition was found. The effects and their interactions of both models are listed in Table 4.

**Table 4:** Results of the linear mixed-effects models of the second experiment with the dependent variables time-to-peak after stimulus onset *t*_D_ and the corresponding peak-to-trough amplitude *a*_PT_

Effect	Time-to-peak after stimulus onset (*t*_D_) *n* = 984, *R*^2^_adj_ = 0.78		Peak-to-trough amplitude (*a*_PT_) *n* = 984, *R*^2^_adj_ = 0.70	
	*F*-Statistic	*p* value	*F*-Statistic	*p* value
*s*	*F*(4, 814.01) = 619.8315	< 0.0001***	*F*(4, 814.01) = 12.9026	< 0.0001***
*c*	*F*(2, 814.01) = 9.0327	0.0001***	*F*(2, 814.00) = 62.3757	< 0.0001***
sf	*F*(10, 814.01) = 67.0932	< 0.0001***	*F*(10, 814.00) = 118.1043	< 0.0001***
*s* x *c*	*F*(8, 814.01) = 4.4766	< 0.0001***	*F*(8, 814.00) = 2.1180	0.0319*
*s* x sf	*F*(40, 814.01) = 3.5557	< 0.0001***	*F*(40, 814.00) = 3.4957	< 0.0001***
*c* x sf	*F*(20, 814.00) = 0.9078	0.5772	*F*(20, 814.00) = 5.5143	< 0.0001***
*s* x *c* x sf	*F*(80, 814.00) = 0.4939	0.9999	*F*(80, 814.00) = 0.5166	0.9998

*s* stimulator type (CRT/high contrast, LCD new/high contrast, LCD new/low contrast, LCD old/high contrast, LCD old/low contrast), *c* condition (sc, 0.6, 0.4), *sf* spatial frequency

Alpha level = 0.05; asterisks indicate the level of significance: *p < 0.05, **p < 0.01, ***p < 0.001

Post hoc comparisons using Dunnett’s tests between the least square means of time-to-peak from the LCD monitors using high and low contrasts and the CRT monitor with high contrast as a control revealed statistically significant delays of the time-to-peak for all monitors and contrasts. A statistically significant difference for the peak-to-trough amplitude was only found between the new LCD monitor with high contrast and the CRT monitor (Table 5).

**Table 5:** Results of a post hoc Dunnett’s test (adjusted degrees of freedom = 814) comparing the least-square means of the linear mixed-effects models of the effect of the stimulator type with the results of the CRT monitor as control (time-to-peak after stimulus onset: 211.9 ± 6.9 ms, peak-to-trough amplitude: 13.1 ± 1.3 ms)

Stimulator	Time-to-peak after stimulus onset (ms)				Peak-to-trough amplitude (µV)			
	Diff. ± SE	95% CI	*t*-value	*p* value	Diff. ± SE	95% CI	*t*-value	*p* value
LCD new, high	128.9 ± 3.5	[120.4, 137.3]	37.31	< 0.0001***	2.6 ± 0.6	[1.2, 4.0]	4.66	< 0.0001***
LCD new, low	125.4 ± 3.5	[116.9, 133.8]	36.29	< 0.0001***	1.0 ± 0.6	[− 0.3, 2.4]	1.84	0.1979
LCD old, high	133.0 ± 3.5	[124.5, 141.4]	38.50	< 0.0001***	0.2 ± 0.6	[− 1.2, 1.5]	0.29	0.9953
LCD old, low	151.8 ± 3.5	[143.3, 160.2]	43.94	< 0.0001***	− 1.2 ± 0.6	[− 2.6, 0.2]	− 2.13	0.1076

Alpha level = 0.05; asterisks indicate the level of significance: *p < 0.05, **p < 0.01, ***p < 0.001

A Tukey–Kramer HSD test did not reveal statistically significant differences between the signal-to-noise ratios calculated from the fitted modified Ricker models for the different combinations of monitor and contrast (Table 6).

**Table 6:** Results of a Tukey–Kramer honestly significant difference test comparing the signal-to-noise ratios (SNR) calculated from fitting the modified Ricker model

Stimulator	SNR ± SD (dB)	Stimulator	SNR ± SD (dB)	Diff. ± SE (dB)	95% CI	*p* value
CRT, high	17.2 ± 8.3	LCD old, high	11.3 ± 6.9	5.9 ± 2.2	[− 0.3, 12.1]	0.0680
CRT, high	17.2 ± 8.3	LCD old, low	11.6 ± 5.0	5.6 ± 2.2	[− 0.6, 11.8]	0.0927
CRT, high	17.2 ± 8.3	LCD new, high	11.9 ± 6.0	5.3 ± 2.2	[− 0.9, 11.5]	0.1272
CRT, high	17.2 ± 8.3	LCD new, low	12.9 ± 6.3	4.3 ± 2.2	[− 1.9, 10.5]	0.3121
LCD new, low	12.9 ± 6.3	LCD old, high	11.3 ± 6.9	1.6 ± 2.2	[− 4.5, 7.7]	0.9439
LCD new, low	12.9 ± 6.3	LCD old, low	11.6 ± 5.0	1.4 ± 2.2	[− 4.8, 7.5]	0.9719
LCD new, low	12.9 ± 6.3	LCD new, high	11.9 ± 6.0	1.0 ± 2.2	[− 5.1, 7.1]	0.9894
LCD new, high	11.9 ± 6.0	LCD old, high	11.3 ± 6.9	0.6 ± 2.2	[− 5.5, 6.7]	0.9987
LCD new, high	11.9 ± 6.0	LCD old, low	11.6 ± 5.0	0.3 ± 2.2	[− 5.8, 6.4]	0.9999
LCD old, low	11.6 ± 5.0	LCD old, high	11.3 ± 6.9	0.3 ± 2.2	[− 5.8, 6.4]	0.9999

Alpha level = .05; asterisks indicate the level of significance: *p < .05, **p < .01, ***p < .001

Visual acuities were estimated from the sweep VEPs for the different combinations of monitors and contrast levels by applying a modified Ricker model either using the conversion factor described in [7] or using a newly calculated conversion factor based on a linear fit between the spatial frequency of the maximum response and the subjective visual acuity measured using FrACT. The differences between estimated and subjective visual acuity were compared to a hypothesized difference of zero using single-sample t tests, which revealed statistically significant differences for all monitor/contrast combinations except for the CRT monitor when using the original conversion factor (Table 7). No statistically significant differences were found for the adjusted conversion formula (Table 7).

**Table 7:** Descriptive statistics of the difference between subjective visual acuity and visual acuity estimated from sweep VEPs using different combinations of monitors and contrasts for stimulation and single sample t-tests comparing the difference to a hypothesized difference of 0 logMAR (*n* = 3 conditions × 6 subjects = 18)

Stimulator	Conversion	Mean difference ± SD (logMAR)	*t*-statistic	*p* value
Original conversion				
CRT, high	VA = 0.23 × sf_max_ + 0.27	− 0.05 ± 0.16	*t*(17) = -1.2447	0.2301
LCD new, high	VA = 0.23 × sf_max_ + 0.27	0.13 ± 0.15	*t*(17) = 3.5129	0.0027**
LCD new, low	VA = 0.23 × sf_max_ + 0.27	0.09 ± 0.10	*t*(17) = 3.8172	0.0014**
LCD old, high	VA = 0.23 × sf_max_ + 0.27	0.16 ± 0.19	*t*(17) = 3.5992	0.0022**
LCD old, low	VA = 0.23 × sf_max_ + 0.27	0.20 ± 0.14	*t*(17) = 6.0349	< 0.0001***
Conversion adjusted per stimulator				
CRT, high	VA = 0.22 × sf_max_ + 0.24	0.02 ± 0.16	*t(*17) = 0.5693	0.5766
LCD new, high	VA = 0.27 × sf_max_ + 0.39	− 0.02 ± 0.15	*t*(17) = − 0.4494	0.6589
LCD new, low	VA = 0.46 × sf_max_ + 0.08	0.02 ± 0.09	*t*(17) = 1.0751	0.2973
LCD old, high	VA = 0.30 × sf_max_ + 0.44	0.00 ± 0.19	*t*(17) = 0.0461	0.9638
LCD old, low	VA = 0.34 × sf_max_ + 0.48	0.02 ± 0.14	*t*(17) = 0.6241	0.5409

The upper half of the table uses the formula from [7] for conversion from the maximum amplitude sf_max_ of the fitted modified Ricker model, the lower half uses conversion formulas calculated individually for each combination of monitor and contrast. Positive mean differences indicate an overestimation, negative ones an underestimation of the subjective visual acuity

Alpha level = .05; asterisks indicate the level of significance: *p < .05, **p < .01, ***p < .001

## Discussion

The obsolescence of CRT monitors requires replacing stimulators used for eliciting VEPs with new monitors. Currently, LCD monitors are the only suitable alternative, however other technologies, like OLED, may become a viable option [23]. So far, the ISCEV extended protocol for VEP methods of estimation of visual acuity recommends ensuring luminance artifacts caused by non-CRT stimulators [9], which can be achieved by reducing the stimulus contrast [23]. However, this may not be possible without falling below the minimum contrast values recommended for VEP [1, 23]. Since LCD stimulators have been shown to result in mostly a delay in the VEP responses [2–4, 23] but seem not to affect the size of the amplitudes [2], we expected no difference between the estimated visual acuity by using LCD or CRT monitors used as a stimulator for the sweep VEP.

The results of the first experiment show statistically significant effects of the monitor type on the time-to-peak after stimulus onset and the peak-to-trough amplitude (Table 1). The mean delay of the time-to-peak after stimulus onset between recordings obtained using the LCD and the CRT monitor was about 60 ms, which is quite high and possibly caused by the relatively old LCD monitor used. Accordingly, statistically significant effects on the time-to-peak after stimulus onset and the peak-to-trough amplitude were found for the monitor/contrast combination in the results of the second experiment (Table 4). Surprisingly, the mean delay of the time-to-peak after stimulus onset of the CRT monitors with high contrast was with up to 151 ms, longer (Table 5) than that of the LCD monitors (with low and high contrast), although one would expect modern monitors to have shorter or even no delays [24, 25]. Additionally, a statistically significant interaction between the spatial frequency and the monitor type was revealed in both experiments, causing an increased time delay for the intermediate spatial frequencies (1.4–10.3 cpd) with LCD stimulation (Fig. 2, top left) in the first experiment and an almost linear increase with the spatial frequencies in the second experiment (Fig. 2, bottom left). This may be explained by the semi-manual cursor placement, which is necessary because the amplitudes are less pronounced at frequencies below and above this frequency band. Another cause might be an input lag resulting from the time required by the monitor to prepare the image data to be displayed. This could be caused by, e.g., internal scaling for non-native resolutions, which may even be present when using the monitor’s native resolution. In the worst case, this leads to nonlinearities of the response timing of the LCD monitor when presenting patterns of low or high frequency [26, 27]. In doubt, the precise duration of the input lag should be measured using a photodiode attached to the display [28] and in case of being constant, the delay could then be subtracted from the respective time-to-peak values. Finally, the higher latencies may also be caused by the different software used for generating the stimuli: whereas in the first experiment, a custom-developed Java-based software was used, in the second experiment, the Python-based PsychoPy was employed. Nevertheless, these differences seem not to affect the estimated visual acuity. The mean peak-to-trough amplitude using the LCD monitor in the first experiment is reduced by about 0.9 µV with a confidence interval from − 1.6 to − 0.2 µV compared to the CRT stimulator, but increased by about 2.6 µV (confidence interval from 1.2 to 4.0 µV) when comparing the new LCD monitor with the CRT monitor (both with high contrast) in the second experiment (Table 5). However, these differences were, despite being statistically significant, within the expected standard deviation from about 0.5 to 7 µV of the P100 amplitude found in the literature [29–31] and therefore probably of no clinical relevance (Fig. 2, right). Interestingly, the results of Nagy et al. [2] suggest a similar reduction in the peak-to-trough amplitude when using an LC display for stimulation. In the first experiment, no statistically significant interaction between monitor type and spatial frequency on peak-to-trough amplitude was found but a tendency to smaller amplitudes at intermediate frequencies (Table 1), whereas in the second experiment, the effect of the interaction of stimulator and spatial frequency was statistically significant (Table 4). It has to be taken into account that the residuals of the models were heteroscedastic and therefore the statistical significance of the effects may be overestimated [32].

In the first experiment, the difference between the subjective visual acuity and that estimated by the second-order polynomial method, or by the modified Ricker function, was not statistically significant from a hypothetical assumed value of 0 logMAR (Table 2). Neither were the variances between CRT and LCD statistically different. Accordingly, the linear mixed-effects models revealed no statistically significant effects of neither the monitor type, the recording cycle, nor their interaction on the difference between subjective and estimated visual acuity for both estimation methods (Table 3).

In contrast in the second experiment, the differences between subjective visual acuity determined using FrACT and the visual acuities estimated using the modified Ricker function along with the conversion formula used in the first experiment were significantly different from the hypothesized difference of 0 logMAR for both, the new gaming LCD monitor and the old LCD monitor, at high and low contrast, but not for the CRT monitor. After using an individually adjusted conversion formula for each monitor/contrast combination, no statistically significant difference from the hypothesized difference of 0 logMAR was found (Table 7). However, one should keep in mind that using the results to calculate the conversion formula used to predict the results is circular reasoning. Nevertheless, it indicates, that using individual established conversion formulas calculated from a sufficiently large number of normative data will minimize the error between true visual acuity and estimated visual acuity.

Table 6 lists the signal-to-noise ratio calculated from the fitted Ricker model for the different combinations of monitors and contrasts. The highest SNR was found for the CRT monitor using high contrast. The LCDs showed lower SNR values. The on average higher amplitudes obtained using LCD monitors (Table 5) indicate that more noise is present when stimulating using LCDs. However, this effect could be caused by the different software used for the stimulus presentation and the lower number of sweeps recorded for averaging compared to the recordings using the CRT monitor. Nevertheless, none of the differences between the SNR values obtained from the different monitor types was statistically significant (Table 6), which corresponds to the findings of Fox et al. [28].

We want to point out the limitations of the current study: We included only healthy participants, so the possible effects of LCD stimulators on patients with reduced visual acuity remain unclear and should be further investigated, especially since we found a statistically significant, albeit not clinically relevant, effect of the monitor/contrast combination on peak-to-trough amplitude and time-to-peak after stimulus onset in the second experiment (Tables 4, 5). Further limitations are that the participants were not stratified by age and that the subjective visual acuity in the first experiment was determined using an eye chart projector, in contrast to the second experiment, where FrACT was used, limiting the accuracy of the estimated value. Finally, this study compared only three specific monitors; therefore, the results are not universally valid.

In conclusion, based on the results of this study, LCD monitors may substitute CRT monitors for presenting the stimuli for the sweep VEP to objectively estimate visual acuity. Newer LCD screens, especially with low response times in the range of 1–2 ms, therefore, allow for a reduction in luminance artifacts at required contrast levels [23], albeit the luminance artifact may not have a large effect on the recorded signals [28]. New technologies like OLED displays [23] may even be better suited, since one the one hand, the onset will be the same for the whole pattern, and on the other hand, LCDs and OLEDs provide a constant luminance level during stimulation, whereas CRTs need a constants pulses to keep the phosphor lit up, causing fast local luminance flashes all the time [28]. Therefore, in contrast to CRTs, LCD and OLED stimulators, e.g., may allow for recording true offset responses [33]. However, caution should be taken when leveraging modern displays for stimulation, since their in-built electronics perform all kinds of sophisticated image-enhancing procedures including color-correction, brightness boosting, contrast enhancement by real-time adjustments of the colors or the backlight, or eyestrain-reducing blue light filtering, with the aim to improve the users’ experience, or to increase the monitors lifetime. This applies in particular to consumer electronics like TVs. Gaming monitors, in addition, use special acceleration drivers, which shut down the backlight, insert black frames (Black Frame Insertion, BFI), or employ variable refresh rates (e.g., Nvidia G-SYNC or AMD FreeSync) to clean the retained image from the eye. Therefore, one should disable any image processing or enhancing functionality in the monitor settings, before using the monitor as stimulator for electrophysiological experiments. Finally, it is advisable to perform a calibration with healthy volunteers using best-corrected and artificially reduced visual acuity and to collect normative data for the employed setup, as always recommended by ISCEV [34], in order to establish an individual conversion formula between the sweep VEP outcome and the estimated visual acuity.

## Supplementary Information

Below is the link to the electronic supplementary material.

Supplementary file1 (PDF 1645 kb)

Supplementary file2 (PDF 1438 kb)

## Data Availability

The datasets generated during and/or analyzed during the current study are available from the corresponding author on reasonable request.
