# Supplementary material for: Comparison of CRT and LCD monitors for objective estimation of visual acuity using the sweep VEP
Source: Doc Ophthalmol. 2022 Jul 5;145(2):133–45. doi: 10.1007/s10633-022-09883-x (PMC9470625; doi:10.1007/s10633-022-09883-x)
Supplement: Supplementary file 1 — Supplementary file1 (PDF 1645 kb) [file 10633_2022_9883_MOESM1_ESM.pdf]

Documenta Ophthalmologica

Comparison of CRT and LCD monitors for objective estimation of visual acuity using the sweep VEP

Torsten Straßer[1,2], Denise Tara Leinberger[1,2], Dominic Hillerkuss[1], Eberhart Zrenner[1,3], and Ditta Zobor[1,2,4]

[1]Institute for Ophthalmic Research, Centre for Ophthalmology, University of Tuebingen, Germany

[2]University Eye Hospital Tuebingen, Centre for Ophthalmology, University of Tuebingen, Germany

[3]Werner Reichardt Centre for Integrative Neuroscience (CIN), University of Tuebingen, Germany

[4]Department of Ophthalmology, Semmelweis University Budapest, Hungary

Corresponding author:

Torsten Straßer

Institute for Ophthalmic Research, University of Tuebingen

Elfriede-Aulhorn-Str. 7

72076 Tuebingen

Germany

torsten.strasser@uni-tuebingen

ORCID: 0000-0001-7725-7961

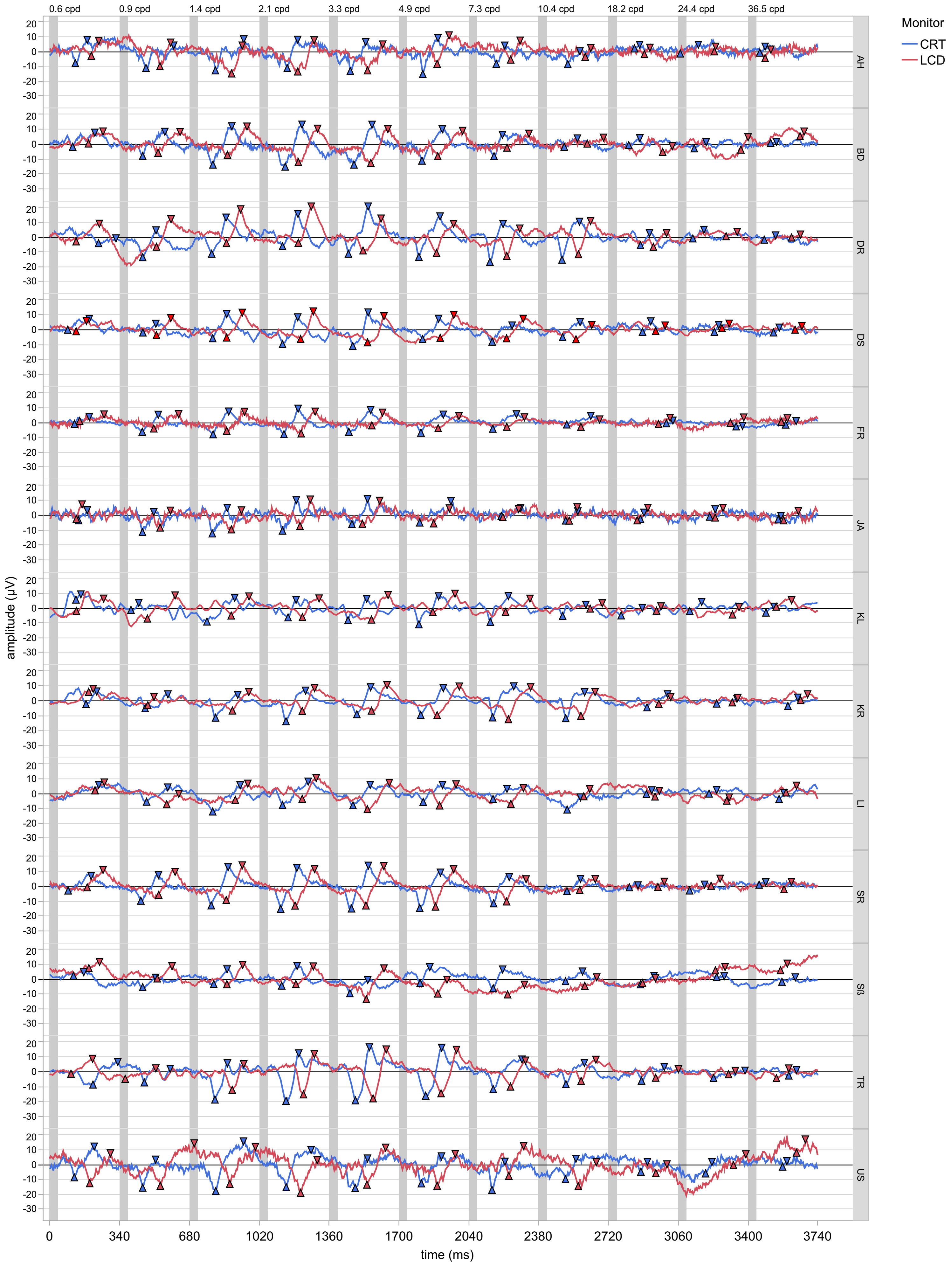

First cycle of the Sweep VEP (50 single sweeps averaged) of the 13 healthy volunteers to repeated stimulation with pattern onset stimulation of increasing spatial frequency (40 ms onset, 300 ms offset, isoluminant, 11 spatial frequencies) presented first, on a CRT (blue), and second, on an LCD (red) monitor. Responses recorded using the LCD monitor are markedly delayed.

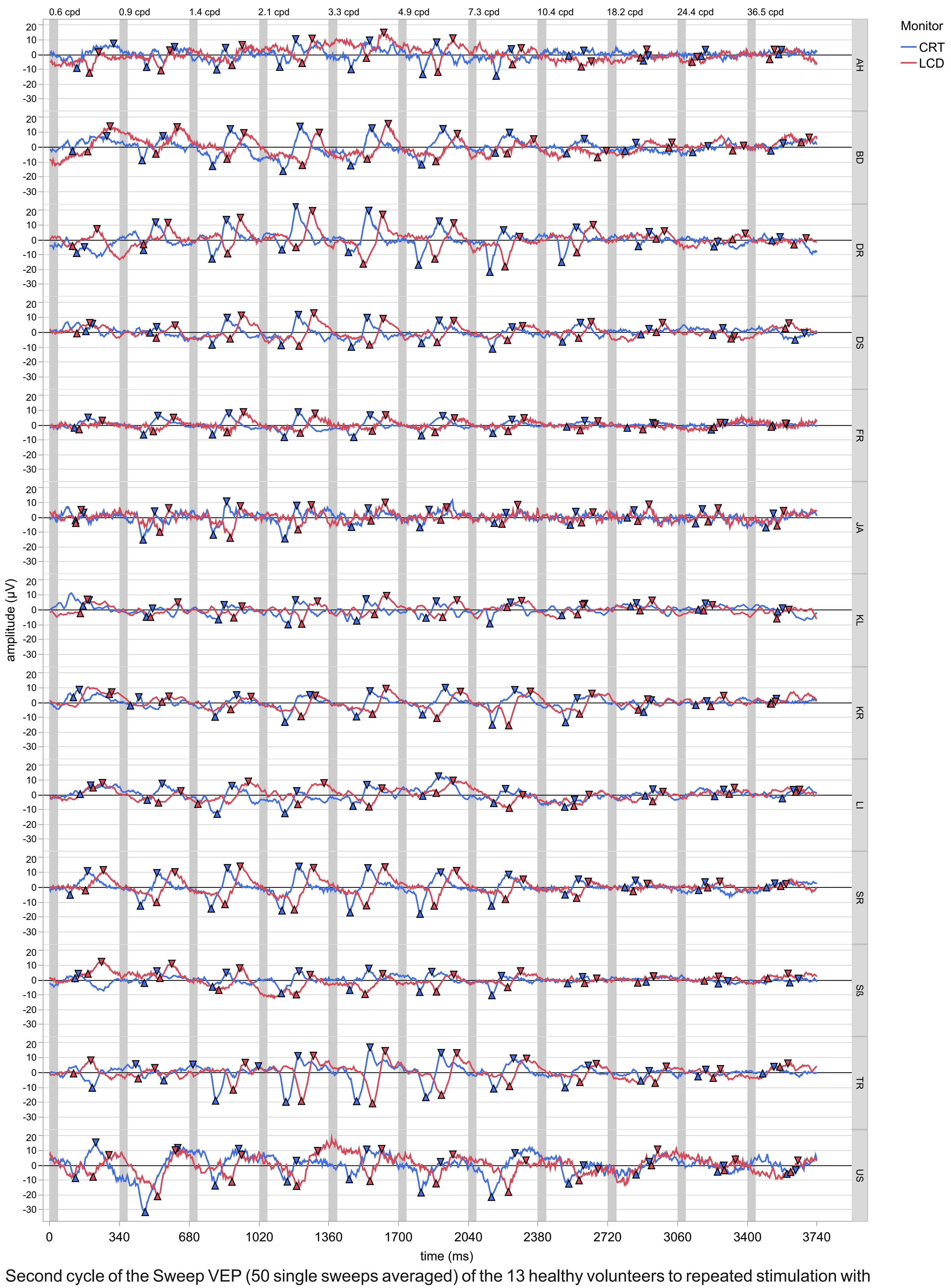

Second cycle of the Sweep VEP (50 single sweeps averaged) of the 13 healthy volunteers to repeated stimulation with pattern onset stimulation of increasing spatial frequency (40 ms onset, 300 ms offset, isoluminant, 11 spatial frequencies) presented first, on a CRT (blue), and second, on an LCD (red) monitor. Responses recorded using the LCD monitor are markedly delayed.
