# Supplementary material for: Comparison of CRT and LCD monitors for objective estimation of visual acuity using the sweep VEP
Source: Doc Ophthalmol. 2022 Jul 5;145(2):133–45. doi: 10.1007/s10633-022-09883-x (PMC9470625; doi:10.1007/s10633-022-09883-x)
Supplement: Supplementary file 2 — Supplementary file2 (PDF 1438 kb) [file 10633_2022_9883_MOESM2_ESM.pdf]

Documenta Ophthalmologica

Comparison of CRT and LCD monitors for objective estimation of visual acuity using the sweep VEP

Torsten Straßer[1,2], Denise Tara Leinberger[1,2], Dominic Hillerkuss[1], Eberhart Zrenner[1,3], and Ditta Zobor[1,2,4]

[1]Institute for Ophthalmic Research, Centre for Ophthalmology, University of Tuebingen, Germany

[2]University Eye Hospital Tuebingen, Centre for Ophthalmology, University of Tuebingen, Germany

[3]Werner Reichardt Centre for Integrative Neuroscience (CIN), University of Tuebingen, Germany

[4]Department of Ophthalmology, Semmelweis University Budapest, Hungary

Corresponding author:

Torsten Straßer

Institute for Ophthalmic Research, University of Tuebingen

Elfriede-Aulhorn-Str. 7

72076 Tuebingen

Germany

torsten.strasser@uni-tuebingen

ORCID: 0000-0001-7725-7961

Artificially reduced visual acuity (0.6)

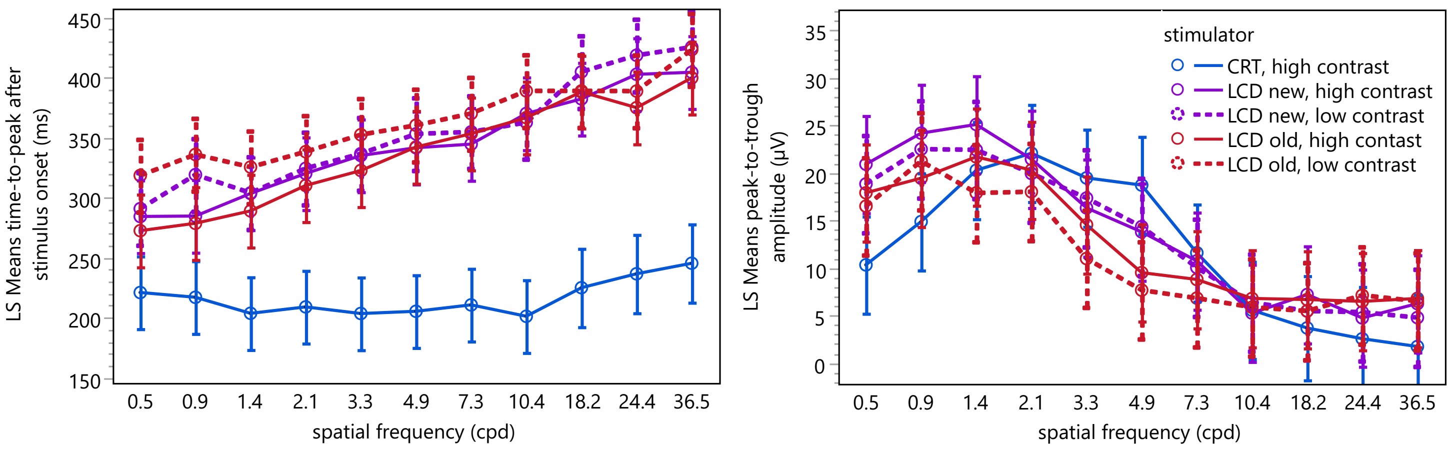

Artificially reduced visual acuity (0.4)

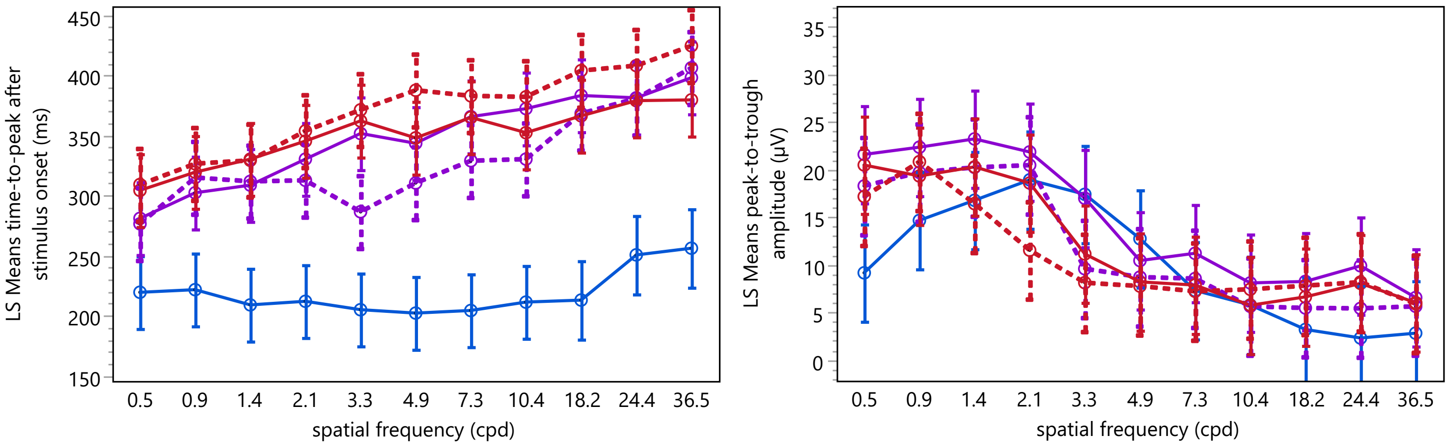

Least-square means and confidence intervals (whiskers) of the linear mixed-effects models (left: time-to-peak after stimulus onset, t_P; right: peak-to-trough amplitude, a_{PT}) for the interaction between the spatial frequency of the stimulation pattern and the stimulator type used for the stimulation (CRT/high contrast: solid blue, LCD old/high contrast: solid red, LCD old/low contrast: dotted red, LCD new/high contrast: solid purple, LCD new/low contrast: dotted purple). Visual acuity was artificially reduced using Bangerter occlusion foils (upper panel: 0.6, lower panel: 0.4)

time (ms)
